# Propionate Decreases Microglial Activation and Alters Phagocytic Machinery in Response to Aggregated Amyloid Beta *in vitro*

**DOI:** 10.1002/alz.087578

**Published:** 2025-01-03

**Authors:** Andrew Gold, Jiangjiang Zhu

**Affiliations:** ^1^ The Ohio State University, Columbus, OH USA

## Abstract

**Background:**

Microglia, the innate immune cells of the brain, are a principal player in Alzheimer’s Disease (AD) pathogenesis. Their surveillance of the brain leads to interaction with the protein aggregates that drive AD pathogenesis, most notably Amyloid Beta (Aβ). Aβ can elicit attempts from microglia to clear and degrade it using phagocytic machinery, spurring damaging neuroinflammation in the process. SCFAs, abundant microbial byproducts of dietary fiber fermentation, are blood‐brain‐barrier permeable molecules that have recently been shown to modulate microglial function. Further, as dietary manipulation can drastically alter SCFA abundances and thus modulate AD microglia, it is crucial to understand SCFAs effects. Propionate, one SCFA, is more poorly characterized than the rest, and thus we attempted to conduct a detailed characterization of propionate’s impact on microglial function in an AD‐relevant *in vitro* experiment.

**Method:**

Using a multi‐omics approach, we characterized the transcriptomic, metabolomic, and lipidomic responses of immortalized murine microglia following 1 hour of Aβ stimulation, as well as characterizing secretion of reactive nitrogen species (RNS) in response to Aβ, using RNA sequencing, LCMS‐based metabolomics, and a fluorescently coupled RNS detector, respectively.

**Result:**

Propionate significantly blunted the early inflammatory response driven by Aβ**,** driving down the expression of many Aβ‐stimulated immune genes, including those regulating inflammation, the immune complement system, and chemotaxis. Further, it reduced the expression of inflammation‐promoting Aβ‐binding scavenger receptors such as cd36 and msr1 in favor of more neutral lpl. Finally, propionate shifted microglial metabolism, altering phospholipid composition and shunting arginine synthesis away from nitric oxide synthesis, which resulted in decreased nitric oxide production.

**Conclusion:**

Altogether, our data demonstrate a modulatory role of propionate on microglia that may dampen immune activation in response to Aβ and potentially improve AD pathogenesis.

